# Inflammatory myofibroblastic tumor of the temporal bone

**DOI:** 10.1590/S1808-86942012000400027

**Published:** 2015-10-20

**Authors:** Gustavo Latorre Samencatti, Felipe Costa Neiva, José Ricardo Gurgel Testa

**Affiliations:** 1 MD. ENT Resident; 2 MD. Otolaryngologist; MSc student - Department of Otorhinolaryngology and Head and Neck Surgery - Medical School of the Federal University of São Paulo - UNIFESP; 3 PhD in sciences - UNIFESP; Adjunct Professor - Department Otorhinolaryngology and Head and Neck Surgery - UNIFESP)

**Keywords:** ear middle, granuloma plasma cell, temporal bone

## INTRODUCTION

The inflammatory myofibroblastic tumor (IMT) is a benign pseudoneoplastic proliferation, of unknown origin, which involves mainly the gastrointestinal tract, liver and lungs. There are just a handful of cases reported in the literature concerning their involvement of the temporal bone, which is the second one with brain invasion.

## CASE PRESENTATION

This study reports on the case of a 59-year old woman complaining of vertigo and sudden hearing loss on her left side lasting for one month. Upon the physical exam, she had an intact tympanic membrane, though hyperemic.

**Figure 1:**
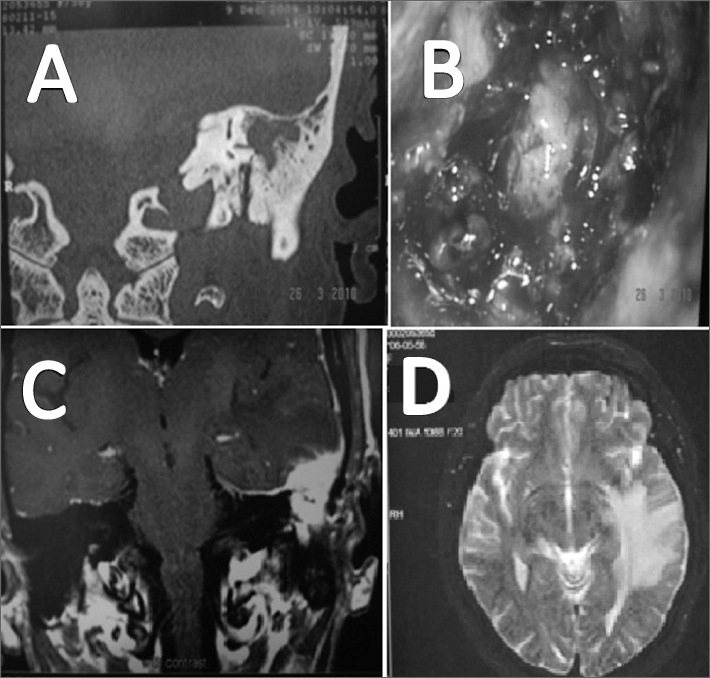
A: Temporal bone CT scan showing a lesion eroding the lateral semicircular canal; B: Tumor aspect during surgery; C: Contrasted skull T1 MRI showing left temporal lobe tumor invasion; D: T2 skull MRI in which we see an important cerebral edema.

We ordered an audiogram, which showed severe mixed hearing loss on the left side. In the left-side temporal bone CT scan we found a soft tissue mass occupying the epitympanic and the mastoid antrum regions, sparing the ossicular chain and the scutum; though eroding the lateral semicircular canal (Figure 1A).

We decided to perform a left-side tympanomastoidectomy, in which we noticed a granulous and infiltrative tumoral invasion of the mastoid antrum. We carried out an excisional biopsy of the lesion (Figure 1B). We did not find signs of the lesion extending to the mastoid tegmen into the intracranial region. The pathology report described it as a case of IMT.

Three months after the tympanomastoidectomy, the patient had intense headaches with an episode of seizure. We ordered an MRI of the skull, which showed the lesion next to the middle fossa meninx, hyperintense in gadolinium uptake, invading the left temporal bone, causing a major adjacent cerebral edema (Figure 1C-D). We treated the patient conservatively with deflazacort for an undetermined amount of time, managing to control tumor growth and reducing the brain edema, which maintains until today.

## DISCUSSION

The IMT is a distinct pseudosarcomatous lesion, of unknown etiology^1^, involving soft tissue and vicera, more frequent in children and young adults^2^. Temporal bone IMT is difficult to diagnose, since it has a large array of differential diagnostic possibilities, such as malignant external otitis, necrotizing bacterial osteomyelitis, fungal osteomyelitis, cholesteatoma, granulomatous diseases, other primary neoplasias and metastases. CT and MRI are of paramount importance to outline the lesion, because of the latter's capacity to destroy the temporal bone and cause intracranial invasion^3^, ^4^.

In 2003, Williamsom et al.^5^ published a review of 10 temporal bone IMT cases. In nine of them they performed total surgical removal and, in one case, it was a subtotal removal. Two cases received steroids, one with a residual tumor and another because of meningeal involvement. In the case with residual tumor, they also used radiotherapy^5^.

In 2008, Montoya et al.^6^ published a case of a 75-year old man with a temporal bone IMT, with skull base invasion and involvement of cranial nerves VI, X, XI e XII. The patient was submitted to tympanomastoidectomy and had dura mater erosion and posterior cranial fossa invasion, and he died afterwards.

There is no consensus regarding the best treatment for temporal bone IMT; nevertheless, we have chosen to surgically remove the tumor whenever possible, saving steroids and radiotherapy for those cases in which it is impossible to operate. The decision to use steroids to control the cerebral edema and the symptoms has proven effective in the short run; however, it does not prevent disease progression. Radiotherapy is controversial, because besides the side effects and an unfavorable prognosis in these patients, it failed to prove survival improvements^5^.

We have seen an increase in the number of these temporal bone tumors in the past 10 years, and its etiology remains uncertain^5^. The reporting of our case is relevant because of its rarity, being the second report of intracranial invasion and the first with more than one year survival with the disease.

## FINAL REMARKS

In summary, temporal bone IMT is rare and represents a very distinct clinical and pathological entity. Despite its benign characteristics, it is usually destructive, involving the middle and inner ears, which can cause bone erosion of the mastoid tegmen and consequent intracranial invasion.
